# IoT powered RNN for improved human activity recognition with enhanced localization and classification

**DOI:** 10.1038/s41598-025-94689-5

**Published:** 2025-03-25

**Authors:** Naif Al Mudawi, Usman Azmat, Abdulwahab Alazeb, Haifa F. Alhasson, Bayan Alabdullah, Hameedur Rahman, Hui Liu, Ahmad Jalal

**Affiliations:** 1 School Department of Computer Science, College of Computer Science and Information System, Najran University, Najran, 55461 Saudi Arabia; 2 Faculty of Computing and AI, Air University, Islamabad, 44000 Pakistan; 3 Department of Information Technology, College of Computer, Qassim University, Buraydah, 52571 Saudi Arabia; 4 Department of Information Systems, College of Computer and Information Sciences, Princess Nourah bint Abdulrahman University, P.O. Box 84428, Riyadh, 11671 Saudi Arabia; 5 Cognitive Systems Lab, University of Bremen, 28359 Bremen, Germany; 6 Jiangsu Key Laboratory of Intelligent Medical Image Computing, School of Future Technology, Nanjing University of Information Science and technology, Nanjing, China; 7 Department of Computer Science and Engineering, College of Informatics, Korea University, Seoul, 02841 South Korea

**Keywords:** Classification and taxonomy, Data acquisition, Data processing, Machine learning

## Abstract

Human activity recognition (HAR) and localization are green research areas of the modern era that are being propped up by smart devices. But the data acquired from the sensors embedded in smart devices, contain plenty of noise that makes it indispensable to design robust systems for HAR and localization. In this article, a system is presented endowed with multiple algorithms that make it impervious to signal noise and efficient to recognize human activities and their respective locations. The system begins by denoising the input signal using a Chebyshev type-I filter and then performs windowing. Then, working in parallel branches, respective features are extracted for the performed activity and human’s location. The Boruta algorithm is then implemented to select the most informative features among the extracted ones. The data is optimized using a particle swarm optimization (PSO) algorithm, and two recurrent neural networks (RNN) are trained in parallel, one for HAR and other for localization. The system is comprehensively evaluated using two publicly available benchmark datasets i.e., the Extrasensory dataset and the Sussex Huawei locomotion (SHL) dataset. The evaluation results advocate the system’s exceptional performance as it outperformed the state-of-the-art methods by scoring respective accuracies of 89.25% and 90.50% over the former dataset and 95.75% and 91.50% over the later one for HAR and localization.

## Introduction

The increasing proliferation of smart gadgets in our daily lives has ushered in a new era of data-driven opportunities, particularly in human activity recognition (HAR) and localization. In today’s technologically driven society, the significance and motivation to work in these fields cannot be overstated^1–3^. The ramifications range from transforming healthcare through remote patient monitoring and early disease detection to increasing security through intelligent surveillance systems^4–6^. Furthermore, the introduction of augmented reality and the expansion of smart cities necessitate precise localization and activity recognition for seamless user experiences and efficient urban planning. The potential to handle real-world difficulties, harness the power of IoT-driven data^7,8^, and contribute to breakthroughs that directly influence society are the exceptional attractions of this research area^9,10^. As we stand at the crossroads of technology and human experience, the pursuit of novel solutions in HAR and localization has the potential to transform our understanding of human behavior and reshape the way we engage with our increasingly pertaining environment^11–13^.

HAR and localization are two independent areas of research that require distinct algorithms and approaches to be worked upon. A lot of research has been performed in these fields using various sensor modalities, including inertial sensors, vision sensors, radars, and wireless fidelity (Wi-Fi). Challa et al.^14^ proposed a multibranch CNN-BiLSTM model for HAR based on data acquired from wearable sensors. Their model combined the benefits of Convolutional Neural Networks (CNNs) with Bidirectional Long Short-Term Memory (BiLSTM) networks. When working with time series data, CNNs extract local features, whereas BiLSTMs excel at handling long-term dependencies by utilizing past and future information. This fact enabled their model to make more accurate predictions.

Furthermore, the model incorporated convolutional kernels of varying sizes, allowing it to capture various temporal local relationships within sequential data. A unique attribute to their model is its ability to fuse locally recognized patterns and long-term sequential data which eventually play a key role in model’s performance. Tan et al.^15^ presented an ensemble learning algorithm (ELA) based on smartphone sensor data for activity recognition. This ELA combined three neural network architectures: a gated recurrent unit (GRU), a CNN stacked on the GRU, and a deep neural network (DNN). In addition to the raw sensor data, the DNN is fed an extra feature vector with 561 time-domain and frequency-domain features. The fully connected DNN combines the predictions of three models (CNN + GRU, GRU, and DNN) for final activity classification. The cohesion of deep learning models and integration of a rich feature set makes the model intelligent enough to efficiently capture complex data patterns and enhance its accuracy. Chen et al.^16^ emphasized the systematic investigation of motion-sensor behavior for smartphone-based human activity recognition. They used cycle detection methods for segregating sensory input sequences, described activities using multiple features, and created tailored and generalized models for activity recognition using classification algorithms. The incorporation of time, frequency, and wavelet-domain information indicates a thorough feature extraction procedure, improving the system’s ability to differentiate between various activities. Data segmentation on the basis of cyclic patterns and multi-domain features make the system adaptable to smartphone sensors based scenarios and enhance the accuracy of the model to recognize diverse activities.

Al-qaness et al.^17^ implemented a deep learning architecture TCN-inception for human activity recognition considering it as a multivariate time series classification problem. To collect long term temporal dependencies, they integrated parallel convolutional layers of temporal convolutional neural networks (TCNs) with inception modules using dialated convolutions and varying kernel sizes. The major contribution of the authors is their model’s ability to capture long term temporal dependencies of the time series data that proved vital for their system’s performance. Xu et al.^18^ proposed a novel framework that implemented a knowledge distillation model that transferred learning from a complex teacher model to a compact student model. The approach was claimed to be efficient for resource limited environments like smartphones that happens to be the key contribution they made.

Cheng et al.^19^ used ultra-wideband (UWB) indoor positioning technology to meet the substantial challenge of identifying and localizing human activities at the same time. They emphasized the significance of this capability in a variety of domains, such as smart healthcare systems, smart homes, human-computer interaction, and robots. Several key components were used in the approach, including improving the positioning accuracy of the UWB system through signal processing and machine learning techniques, as well as using machine learning methods such as support vector machines, artificial neural networks, and hidden Markov models to recognize five types of human activities based on UWB range measurements. The integration of UWB with advanced signal processing makes the system a good choice for real time monitoring and response applications due to its ability to recognize the human activities along with their context at the same time. Bracken et al.^20^ used smartphone sensors to address continuous and real-time health assessment concerns. They recognized that the standard health evaluation procedures are infrequent, expensive, and necessitate specialist equipment. The idea was to use passive and unobtrusive data acquired by smartphone sensors to continuously analyze people’s health. The initial step was to determine the context in which the smartphone was used, as this context considerably impacts sensor data interpretation. The DeepSense deep learning technique is employed for feature learning and context categorization. Their system is specially significant in terms of scalability and context-aware activity recognition using smartphone data that is difficult to interpret. But DeepSense proved its capability by outperforming the state-of-the-art systems. Nakamura et al.^21^ presented a method for multi-stage activity inference developed by Ubi-NUTS Japan. The method aimed to identify a user’s mode of locomotion and transportation using mobile device sensors, emphasizing the Sussex-Huawei Locomotion-Transportation (SHL) dataset. In this dataset, inertial sensor data from a smartphone was used to recognize 8 forms of locomotion and transport activities. The method used a multi-stage strategy, breaking down the 8-class classification problem into sub-problems based on activity similarity. The classification procedure incorporated feature extraction and the usage of four distinct types of classifiers developed by the random forest algorithm. The key feature of their system is breaking down the main activity classification problem into sub problems based on the activity similarity. Although it enhances the system’s accuracy but might have a higher time and space complexity.

Ronald et al.^22^ designed a system that set its basis on inception-ResNet model. The opted to minimize the resource usage and meanwhile enhacing the predictive accuracy of the HAR model. By leveraging the residual connections that they integrated with inception module, the system was able to outperform deep models including CNN, CNN-LSTM, and stacked and vanilla LSTM. The system architecture allows easy scalability with the addition of more inception modules without much compromise on the performance. Paulose et al.^23^ also utilized smartphone cameras along with traditional inertial sensors including accelerometer and gyroscope. They first detected the human body using masked R-CNN^24^ and then preprocessed the image using facial image threshing. Utilizing these preprocessed images along with sensory data, they trained multiple deep models including VGG, inception, ResNet, and Efficient net. A significant high point of their system is a thorough preprocessing pipeline that enhances the robustness of their system and also contributes to a good activity recognition accuracy.

The system presented in this article uses a multi-stage technique to be immune to signal noise and efficiently recognizes human activities and their locations. It commences by applying a Chebyshev type-I filter to denoise the input sensor data. The denoised data is then passed through windowing that segments it into smaller, more manageable chunks for robust analysis. This system’s parallel processing branches, which work in tandem to extract significant features for both activity recognition and human localization, are one of its standout features. The Boruta algorithm is used to improve feature selection while reducing computing overhead. It facilitates the identification of the most informative features among those extracted, allowing the system to specialize in the most relevant data for HAR and localization. Then a particle swarm optimization (PSO) refines sets of input feature vectors iteratively to obtain one optimal feature vector against each set. The core of the system is the utilization of two Recurrent Neural Networks (RNNs) that function in parallel. One RNN is dedicated to HAR, while the other works on localization. This parallel processing strategy enables the system to accomplish both operations simultaneously, enhancing its efficiency. The major novelty of the proposed system is its synchronization ability of HAR and human localization predictions. By leveraging this, it can easy be implemented in real-time applications providing high accuracy both of the mentioned tasks. While in comparison with the previously discussed state-of-the-art (SOTA) methods, the proposed system is able to outperform them by leveraging both machine learning and deep learning approaches and setting its basis on diverse hand-crafted features, SOTA feature selection and optimization algorithms, and sequential deep learner algorithm RNN.

The major scientific contributions of this study are as follows:

1. A Chebyshev type-I filter is utilized as a signal processing technique that removes noise from the input data, making the system noise-resilient and more reliable.
2. Another noteworthy contribution is the use of parallel processing branches to extract features for activity recognition and human localization simultaneously. This method increases the system’s efficiency by allowing it to execute both operations concurrently without sacrificing computing performance.
3. The Boruta algorithm is employed for feature selection, which establishes the most informative features among those extracted from the data. As a result, the system optimizes its resource allocation and focuses on the data items most relevant for HAR and localization.
4. PSO is implemented as an optimization approach to fine-tune the data before sending it to the classification model for training. This ensures that the model is trained on the best available features to produce the best possible results.
5. The utilization of two RNNs operating in parallel, one for HAR and the other for localization, contributes significantly to the system’s architecture. RNNs are well-suited to analyzing sequential data, and using them in parallel allows the system to address both activity recognition and localization concurrently. This technique not only increases efficacy but also provides a holistic understanding of user’s activity and location.

The rest of the article is structured as follows: Sect. 2 describes the nitty-gritty of the proposed system, while Sect. 3 gives a thorough analysis of the system through experimentation results over two publicly available benchmark datasets i.e., the Extrasensory dataset and SHL dataset. It also gives a comparison of the proposed system with the state-of-the-art methods. Finally, Sect. 4 concludes the article and provides future directions to work in the respective research area.

## The proposed system

**Fig. 1:**
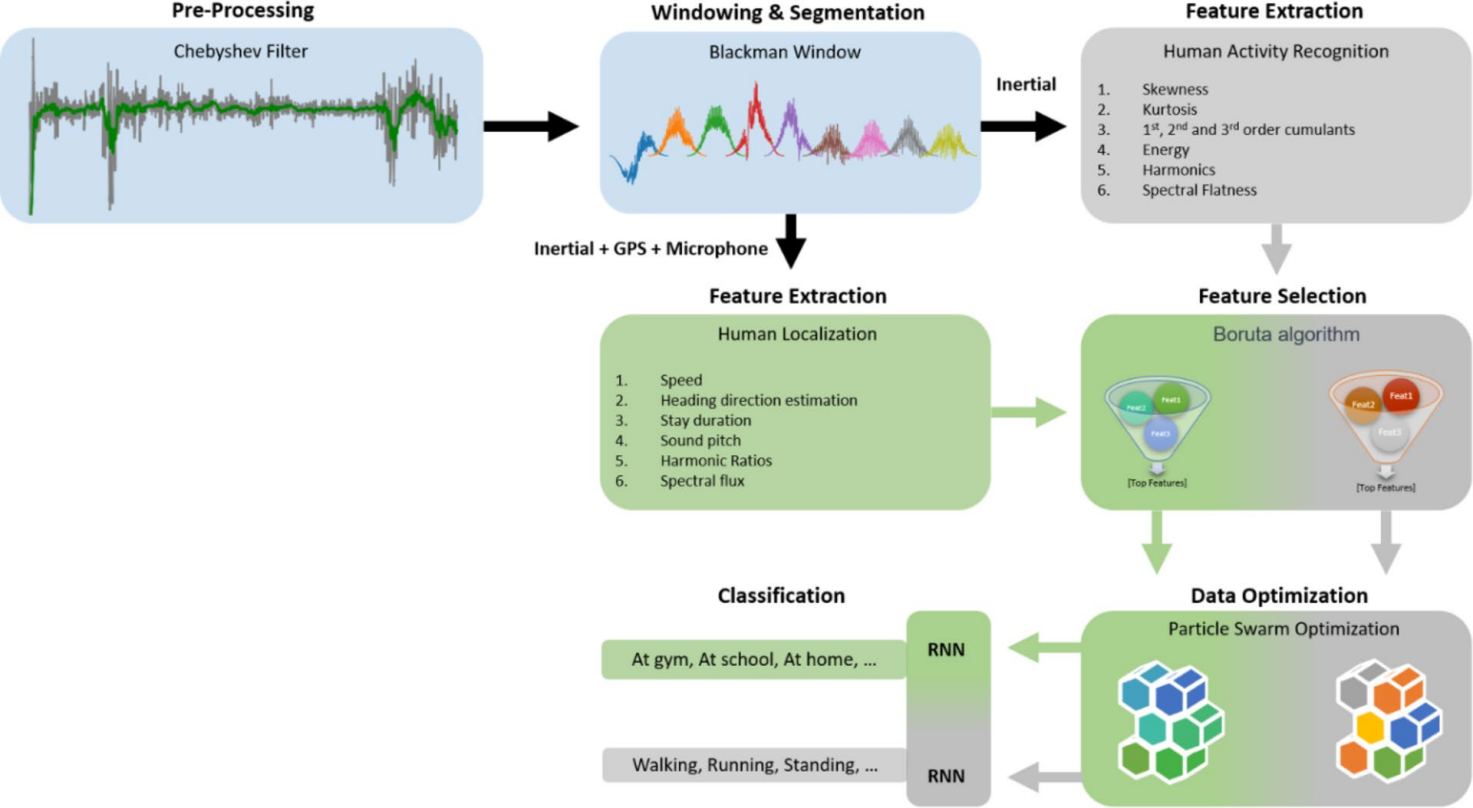
The proposed architecture for HAR and human localization.

The proposed system deals with recognizing the activities performed by humans and identifying the location of the human at which he is performing the respective activity. In this article, identifying the human location (at home, at beach, at gym and so on) is also considered a classification problem. Due to the dependency of both of the mentioned problems on different features and sensors, the system solves them in parallel. It begins by denoising the input signal using a Chebyshev type-I filter and then splits the signal into Blackman windows while each window contains 1.5 s long part of the signal. For the feature extraction, the system gets divided into two parallel branches where it extracts multiple hand-crafted features for HAR and human localization. Features for HAR include skewness, kurtosis, comulants, harmonics, energy, and spectral flatness, while the features for human localization include speed, heading direction, stay duration, sound pitch, harmonic ratios, and spectral flux. To ensure the system’s best performance, a feature selection based on Boruta algorithm is carried out that generates the best set of features to represent the data. After that, data is further optimized by employing a PSO that generates one best feature vector against an input feature population of 10 feature vectors. This optimized data is then fed to two parallel RNNs for the final predictions of the activity and location. The architecture of the system is illustrated in Fig. 1.

### Pre-processing

**Fig. 2:**
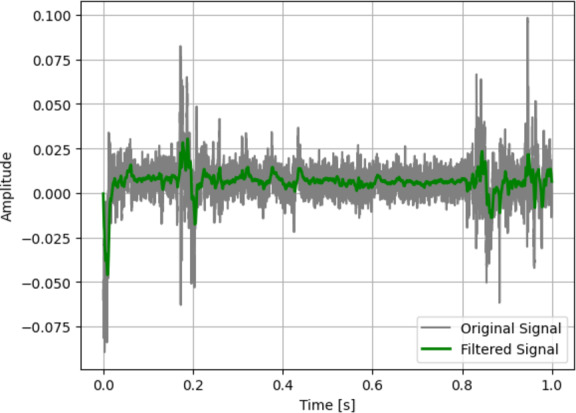
Signal pre-processing using Chebyshev type-I filter.

The pre-processing is crucial while working with IoT data acquired from smartphone and smartwatch sensors. Noisy signals lead to bad features that affect the system’s overall performance. In this study, a Chebyshev^25^ type-I filter is utilized to denoise the signal data. It is an elliptical filter that offers a steeper roll-off and a faster transition between the passband and stopband than the Bessel and Butterworth filter. These features enable it to enhance the signal-to-noise ratio of the input signal by distinguishing between desired components of the signal and noise with precision and minimising the overlap between the unwanted frequency components and actual signal. An illustration of a raw and a pre-processed signal is shown in Fig. 2.

### Windowing

A Blackman window^26^ is utilized for the windowing of the denoised signal. The main attributes of the Blackman window are its wide main lobe, symmetry, and tapering. Wide main lobe and tapering ensure a better frequency resolution that is specifically beneficial for spectral features that play a significant role in both HAR and localization. By taking the window size as a hyper parameter and tuning it for the best results, a window size of 1.5 s proved optimal. The governing equation of the Blackman windowing is as follows:

1 $$\:{W}_{n}=0.42-0.5cos\left(\frac{2\pi\:n}{N-1}\right)+0.08cos\left(\frac{4\pi\:n}{N-1}\right)\:$$

Where $\:{W}_{n}$ is the value of the signal at nth sample, and $\:N$ is the total number of samples in the respective window. The features are extracted from each window independently and then concatenated to form the final feature vector.

### Feature extraction for HAR

The features play a vital role in the performance of an artificially intelligent system. Good features can enhance the reliability and efficiency of the system manifold and vice versa. This study’s HAR features were extracted while utilizing only the inertial sensors data from both smartphone and smartwatch depending upon availability. The features extracted for HAR are as follows:

- Skewness: It assesses the distribution of the input signal and determines whether the data is skewed to the left (negative skewness), to the right (positive skewness), or normally distributed.
- Kurtosis: A signal distribution with positive kurtosis (leptokurtic) has heavier tails and a sharper central peak, whereas a negative kurtosis (platykurtic) distribution has lighter tails and a flatter central peak. While zero kurtosis indicate tail and a peak comparable to the normal distribution.
- 1st order comulant: It is the central moment of data that measures the location and quantifies a random variable’s expected value.
- 2nd order cumulant: The second central moment of data quantifies data dispersion around the mean.
- 3rd order comulant: The third central moment of data provides information about the deviation of data distribution from the normality.
- Energy: A high-energy signal has more substantial and pronounced features, whereas a low-energy signal is weaker and less pronounced.
- Harmonics: A signal’s harmonics provide vital frequency composition and structure information. They are integer multiples of the signal’s fundamental frequency and indicate the presence of periodic components in it.
- Spectral Flatness: A signal with a high spectral flatness is primarily noise-like, whereas a signal with a low spectral flatness is considered harmonically rich.

**Fig. 3:**
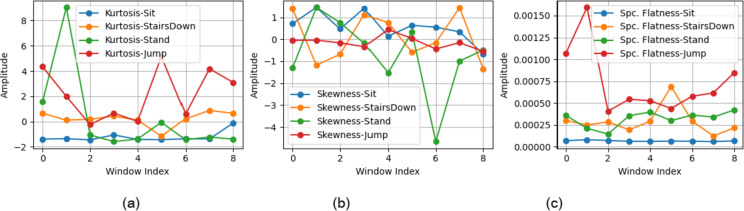
Comparison of HAR features for different activities.

A comparison of the HAR features extracted for various activities is shown in Fig. 3.

### Feature extraction for human localization

For the human localization task, completely different features are required to represent the data. The sensors used in this module include inertial sensors, a global positioning system (GPS), and a microphone. Following are the features that are extracted to represent the location of the user:

- Speed: It is the speed by which the user is moving at a particular location. It is calculated using the longitudes, latitudes, and their respective time stamps provided by GPS data. The covered distance is divided by the consumed time to compute the movement speed.
- Heading Direction: Accurate estimation of the heading direction requires inertial sensor data combined with the GPS data. It is calculated using both modalities independently, and then their mean is accepted as the final estimate.
- Stay Duration: It is calculated using the longitude and latitude information and their respective time stamps provided by the GPS. The time for which the subject’s coordinates remain intact represent the stay duration.
- Sound Pitch: Sound pitch is calculated using the microphone data and proves extremely useful to get information about the user’s surroundings. For instance, if the microphone has caught the sound of heavy breaths, the user might be at the gym.
- Harmonic Ratios: They convey essential information about the spectral content and harmonic structure of the input signal while representing the relative strengths harmonics in the frequency spectrum.
- Spectral Flux: It quantifies the rate of change in the spectral content of a signal across time. It measures the rate at which the energy distribution in the frequency spectrum varies, reflecting dynamic changes in the signal.

**Fig. 4:**
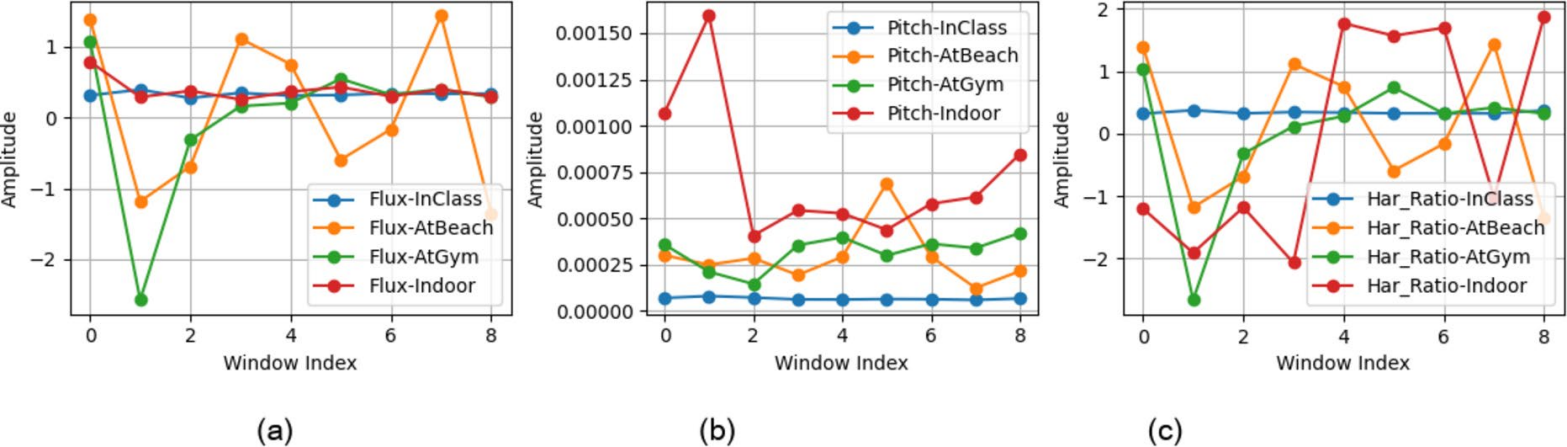
Comparison of localization features for different locations.

A comparison of the localization features extracted for various locations is shown in Fig. 4.

### Feature selection

Feature selection enhances the system’s performance by filtering out the features that prove to be a liability to the system. For this purpose, a Boruta^27^ algorithm is implemented that begins by creating a shadow of the original feature set. Then a random forest classifier is used to determine the rank of each feature based on its importance value. The Z score works as the importance value obtained by dividing the average accuracy by the standard deviation of the feature. The process is iterated several times to achieve the stopping criterion. The Boruta algorithm is given below:

**Algorithm 1:**
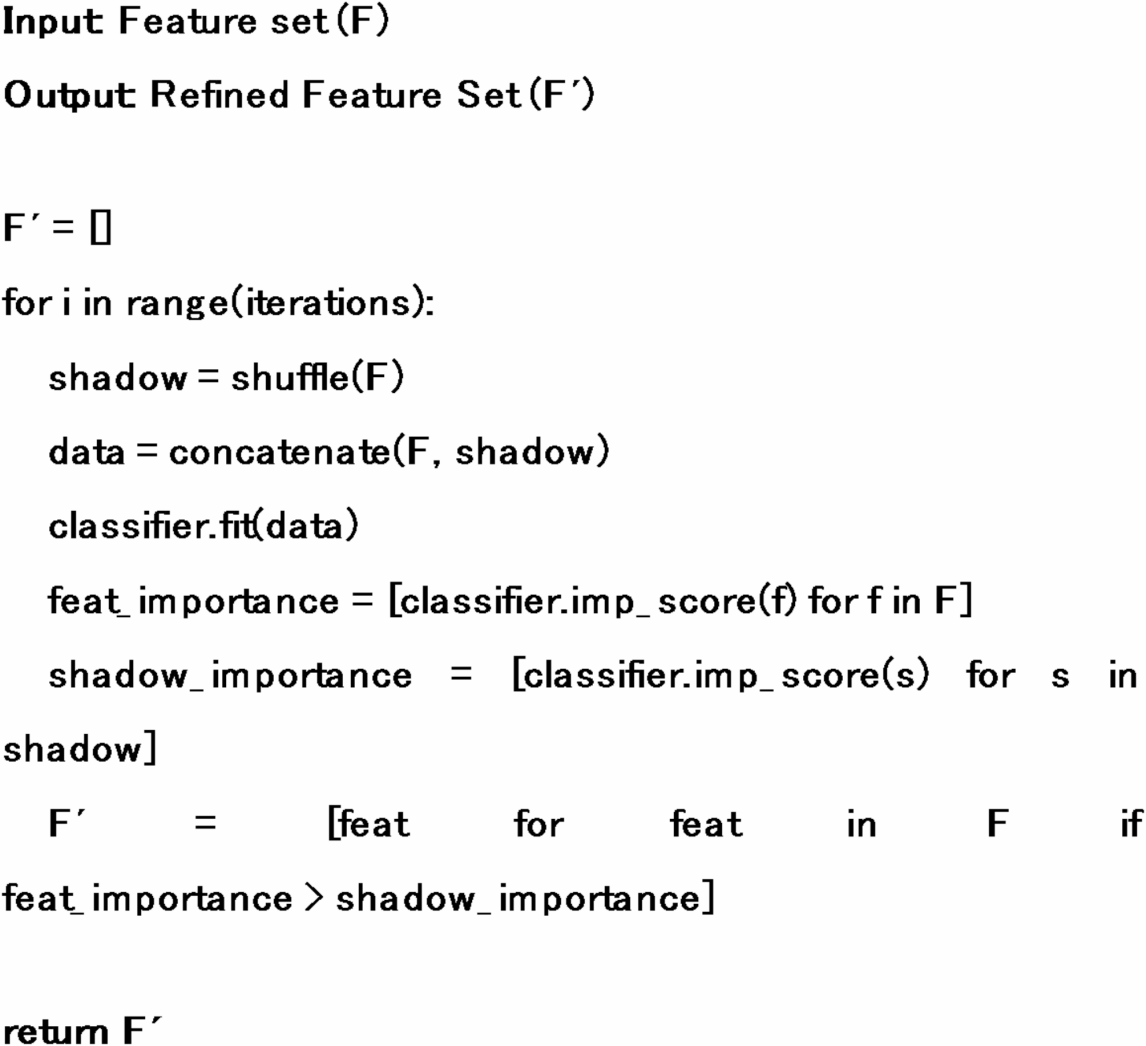
Feature Selection based on the Boruta algorithm.

### Data optimization

Data optimization proves to be a game changer in an artificially intelligent system. In the proposed system, we use a particle swarm optimization^28^ (PSO) to optimise our data. It starts by splitting the original dataset into batches, each consisting of 10 feature vectors. A batch is processed such that the internal contents of the feature vectors are intermixed, and hybridized feature vectors are generated. The selection of the global best feature vector is the core of PSO that tends to maximize the objective function that checks the Z score of each hybridized feature vector one by one. The vector producing the highest Z score is considered the local best for the current iteration. After executing the predefined number of iterations, PSO discovers the global best feature vector. Against each batch, one global best feature vector is produced that is used for the final training of the classification model. The detailed algorithm of PSO is given below:

**Algorithm 2:**
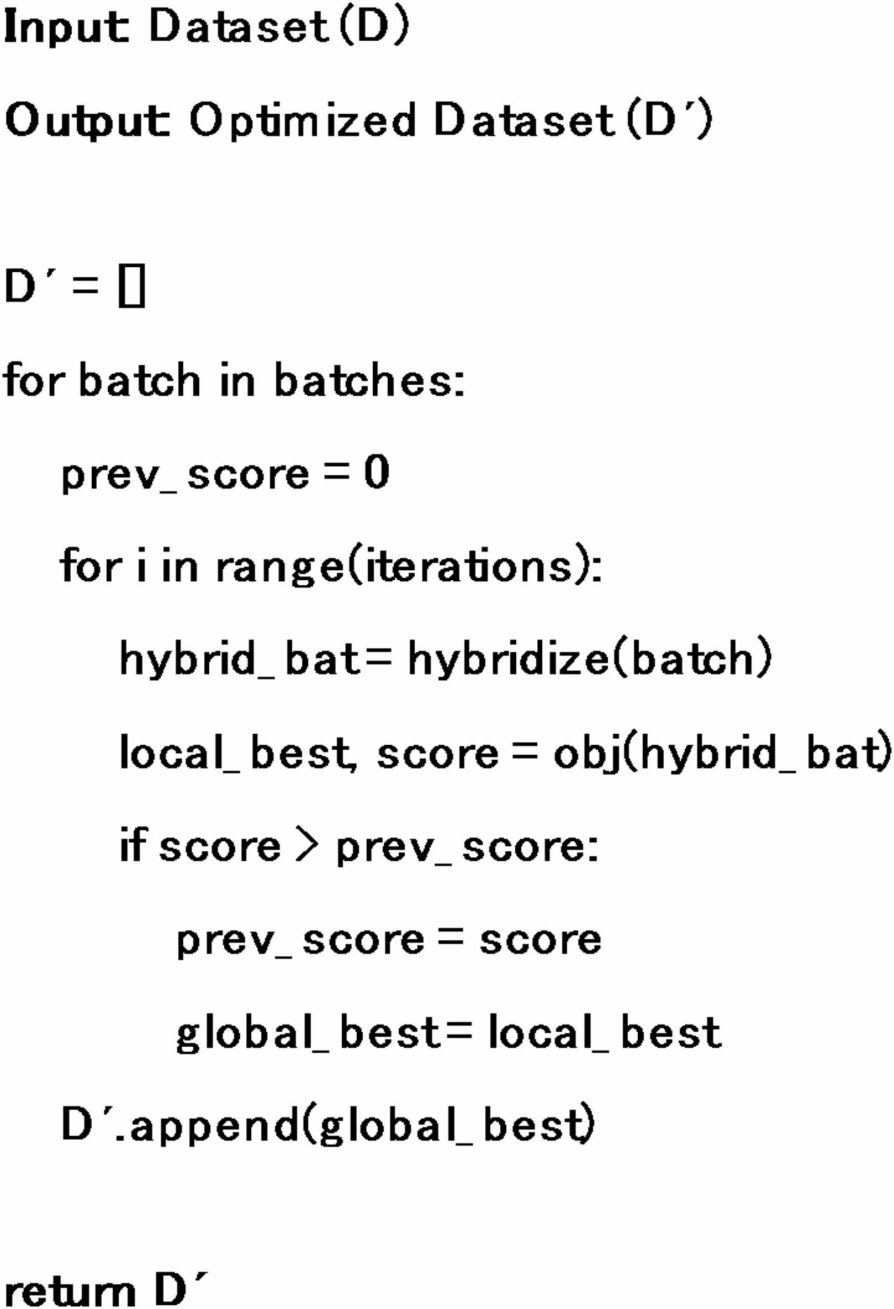
Data Optimization based on PSO.

### Activity classification

A recurrent neural network^29^ (RNN) proves to be a prime choice when sequential data is under consideration. The IoT data collected for the HAR branch of the proposed framework consists of only inertial sensor data that is sequential in nature. Each time step is taken as a feature and fed to RNN for training. Throughout the training process, RNN learns the patterns that define the performed activity, and by comparing the learned patterns, it classifies the respective activities. The structure of RNN utilized by the proposed system is shown in Table 1.

**Table 1:** RNN architecture for HAR over the extrasensory dataset.

Layer (type)	Output Shape	Parameters	Activation	Optimizer	Learning Rate
SimpleRNN	(None, 64)	4224	leaky ReLU	Adam	10^− 4^
Dropout	(None, 64)	0	–		
Dense_1	(None, 64)	4160	leaky ReLU		
Dense_2	(None, 8)	520	Softmax		

Total parameters: 8904 (34.78 KB).

Trainable parameters: 8904 (34.78 KB).

Non-trainable parameters: 0 (0.00 Bytes).

*Dense_2 layer had 4 units in case of SHL.

### Location classification

Location identification, a classification problem in this study, utilized the sequential data acquired from the inertial sensors, GPS, and microphone. Analogous to HAR, location classification also employs an RNN but with a different architecture, which is shown in Table 2.

**Table 2:** RNN architecture for human localization over the extrasensory dataset.

Layer (type)	Output shape	Parameters	Activation	Optimizer	Learning rate
SimpleRNN	(None, 128)	16,640	leaky ReLU	Adam	10^− 3^
Dropout	(None, 64)	8256	–		
Dense_1	(None, 32)	2080	leaky ReLU		
Dense_2	(None, 8)	264	Softmax		

Total parameters: 27,240 (106.41 KB).

Trainable parameters: 27,240 (106.41 KB).

Non-trainable parameters: 0 (0.00 Bytes).

*Dense_2 layer had 6 units in case of SHL.

## Experimental results

### Experimental datasets

The ExtraSensory dataset is an extensive collection of sensor data from 60 individuals, each with a unique identification tag, across a series of intervals, usually lasting one minute. The dataset comprises readings from builtin sensors of users’ cell phones and smartwatches. A watch accelerometer, gyroscope, magnetometer, location information, audio, and a watch compass are among the sensors used in the dataset. The dataset is related to human activities such as sitting, lying down, standing, biking, running, strolling, and climbing and descending stairs. While indoor, at home, at school, at office, outside, in class, at gym, and at beach were among the settings used for this study.

The Sussex-Huawei Locomotion (SHL) dataset is a freely accessible dataset created to support the design and assessment of algorithms for localizing and recognizing human activities. Data from different sensors, including accelerometer, gyroscope, magnetometer, and GPS, are included in the dataset. The dataset consists of labelled data collected from four smartphones placed invarious places, including the pocket, hand, torso, and bag. The dataset includes data on sitting, standing, walking, and running activities as along with indoor, outdoor, in bus, in train, in subway, and in car locations.

### Evaluation on the extrasensory dataset

**Fig. 5:**
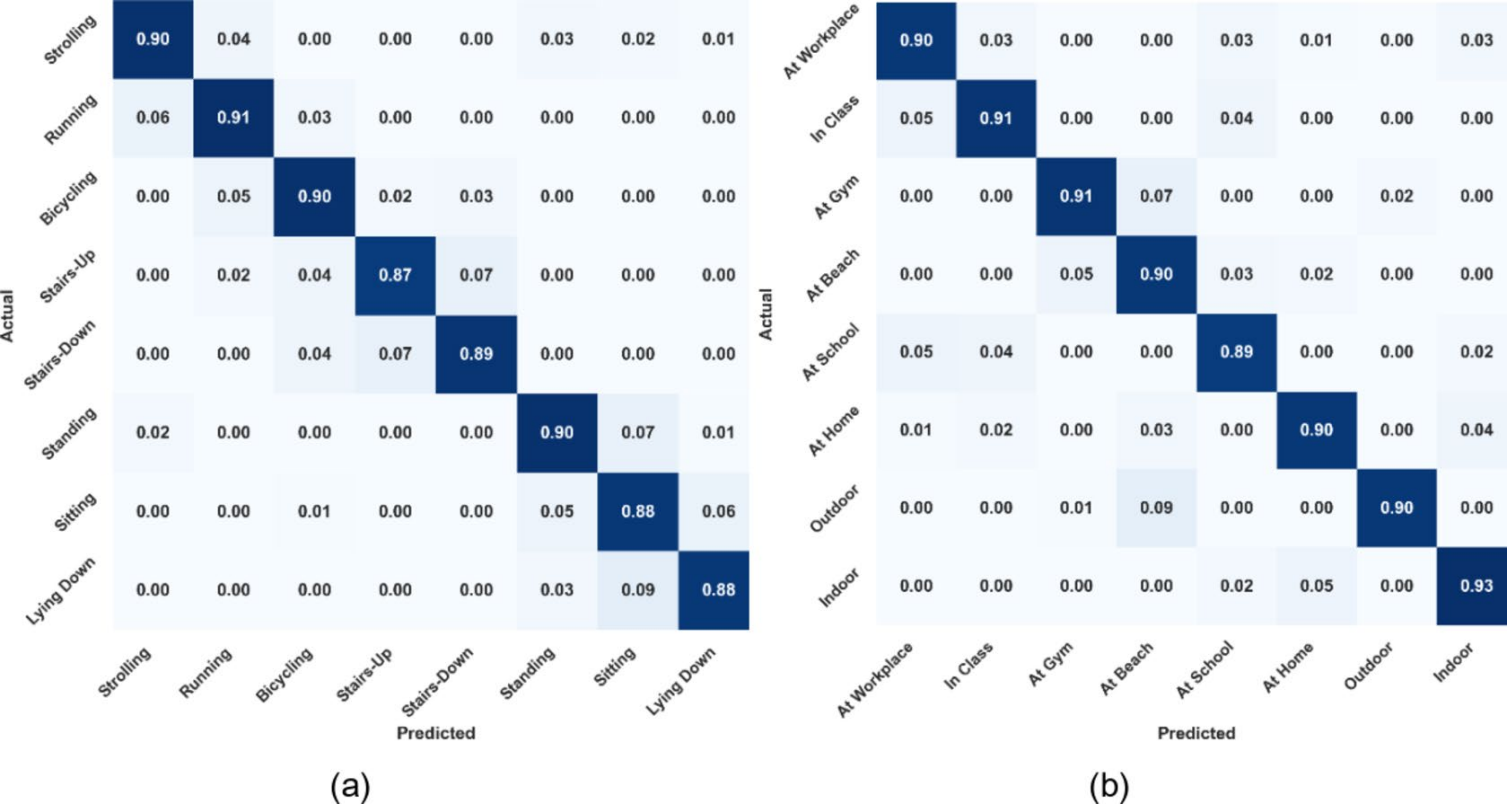
(**a**) Confusion matrix for HAR on Extrasensory dataset (**b**) Confusion matrix for human localization on Extrasensory dataset.

The Extrasensory dataset’s eight human activities and eight locations were used to assess the proposed system thoroughly. Figure 5(a) provides a confusion matrix for the model’s performance for activity recognition over the Extrasensory dataset. Thus, “Running” was the activity with the highest recognition confidence level, i.e., 91%. The model’s confidence level for the prediction of “Stairs-Up” was at its lowest, 87%. Using an RNN classifier, the activity categorization achieved an overall score of 89.25%. A confusion matrix was also plotted for the location classification, whose statistics are shown in Fig. 5b. The highest recognition accuracy achieved by “Indoor” was 93%. While the lowest recognition accuracy was 89% scored for “At School” location. The mean accuracy of localization was 90.50%.

**Fig. 6:**
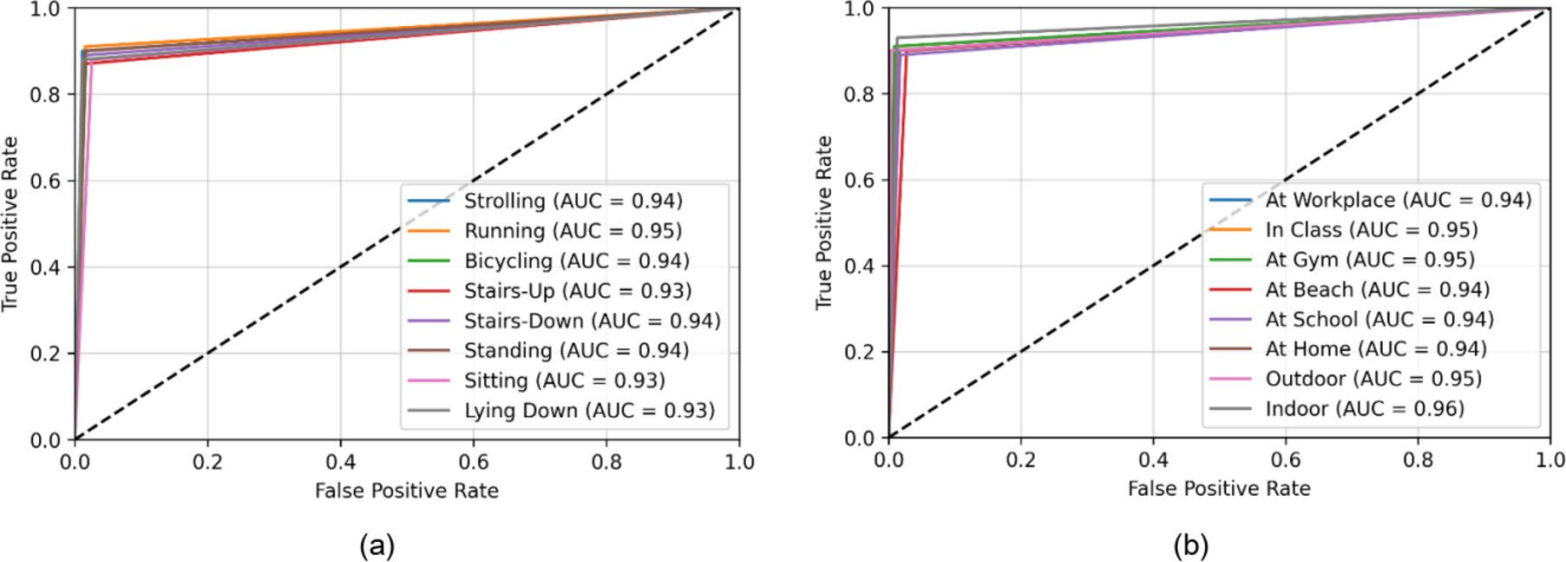
(**a**) ROC curves for HAR on Extrasensory dataset (**b**) ROC curves for human localization on Extrasensory dataset.

**Fig. 7:**
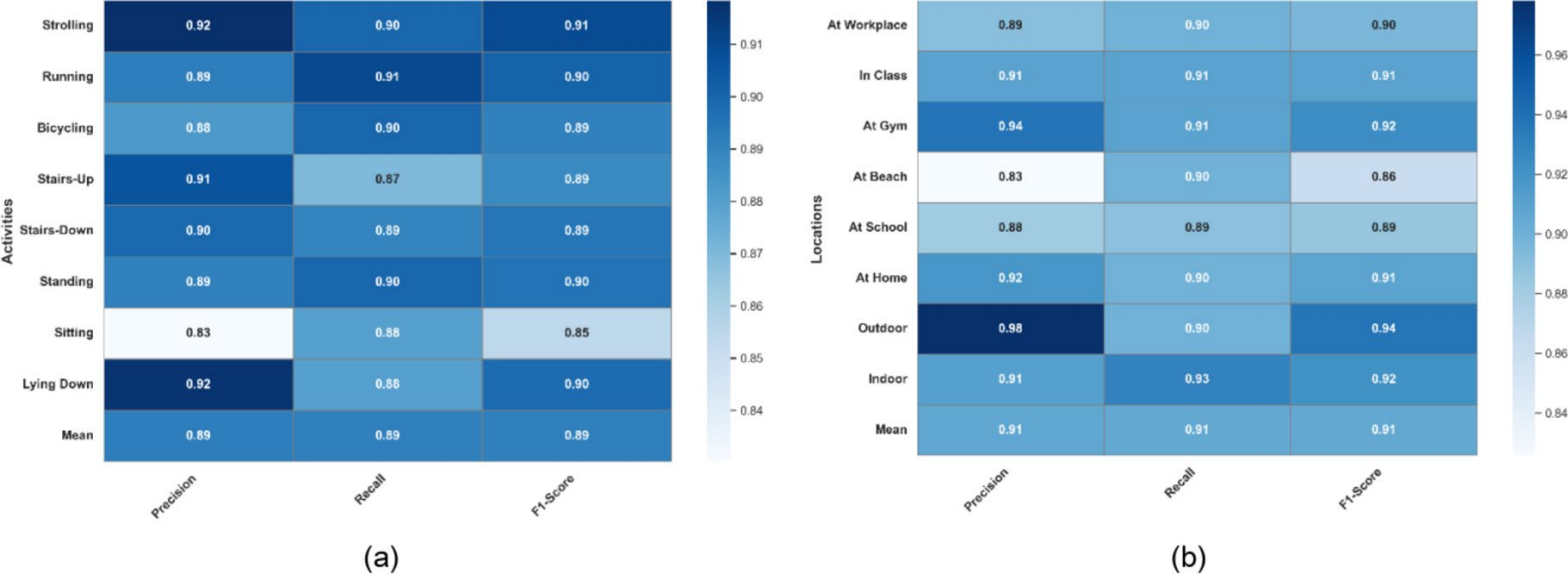
(**a**) Classification report for HAR on Extrasensory dataset (**b**) Classification report for human localization on Extrasensory dataset.

The receiver operating characteristic (ROC) curves are also plotted for the Extrasensory datset regarding HAR and human location that are shown in Fig. 6a and b respectively. The area under the curves (AUC) of activity recognition and localization is consistently above 0.90 that indicates a reliable performance of the system over the mentioned dataset. Furthermore, the classification reports for activity recognition and localization can be examined in Fig. 7(a) and 7(b) respectively. For HAR, the maximum precision is demonstrated on strolling and lying down activities i.e., 92% contributing to a mean precision of 89% while strolling also scored highest F1-score of 91% while mean F1-score was also recorded to be 89%. Regarding localization, 98% precision was recorded for outdoor location which was the highest among others while the F1-score for the recognition of the same location was 94% which was still higher than all other locations. The mean precision and F1-score was recorded to be 91% each.

### Evaluation on SHL dataset

**Fig. 8:**
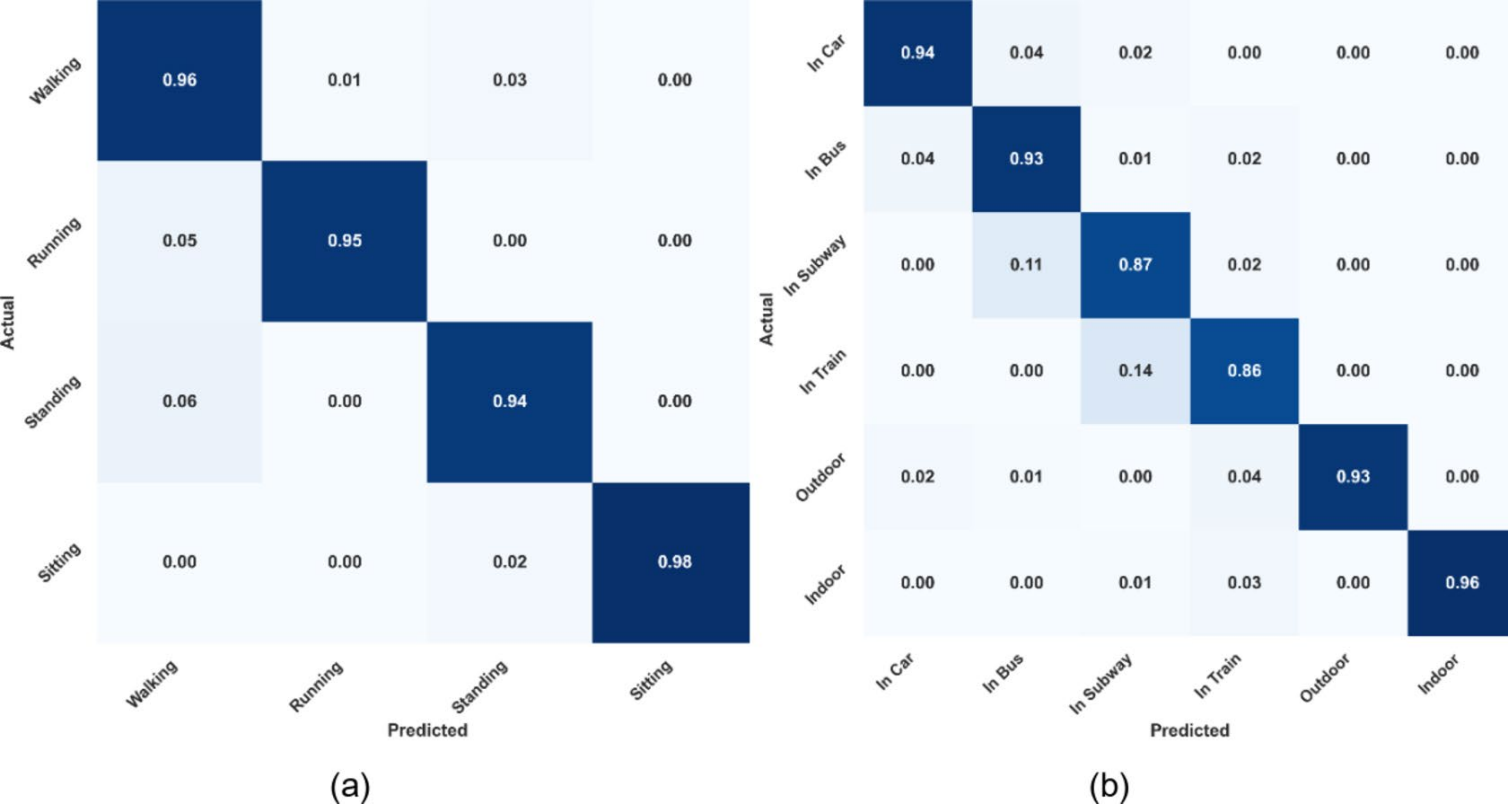
(**a**) Confusion matrix for HAR on SHL dataset (**b**) Confusion matrix for human localization on SHL dataset.

The SHL dataset includes 4 human activities at 6 different locations. The model has been thoroughly evaluated for identifying the activities over the SHL dataset, and the confusion matrix is shown in Fig. 8(a). The activity that was predicted with the highest accuracy i.e., 98%, was “Sitting” while the activity with the lowest accuracy i.e., 94% was “Standing.” While the system had an average HAR accuracy of 95.75% on the SHL dataset. The confusion matrix in Fig. 8(b) illustrates the promising outcomes from the localization over the SHL dataset. The location “Indoor” was correctly predicted 96% of the time out of six distinct locations, which was the highest accuracy rate. While the “In Train” location had the lowest recognition accuracy i.e., 86%. To show its reliability, the algorithm consistently predicted every class with an average accuracy of 91.50%.

**Fig. 9:**
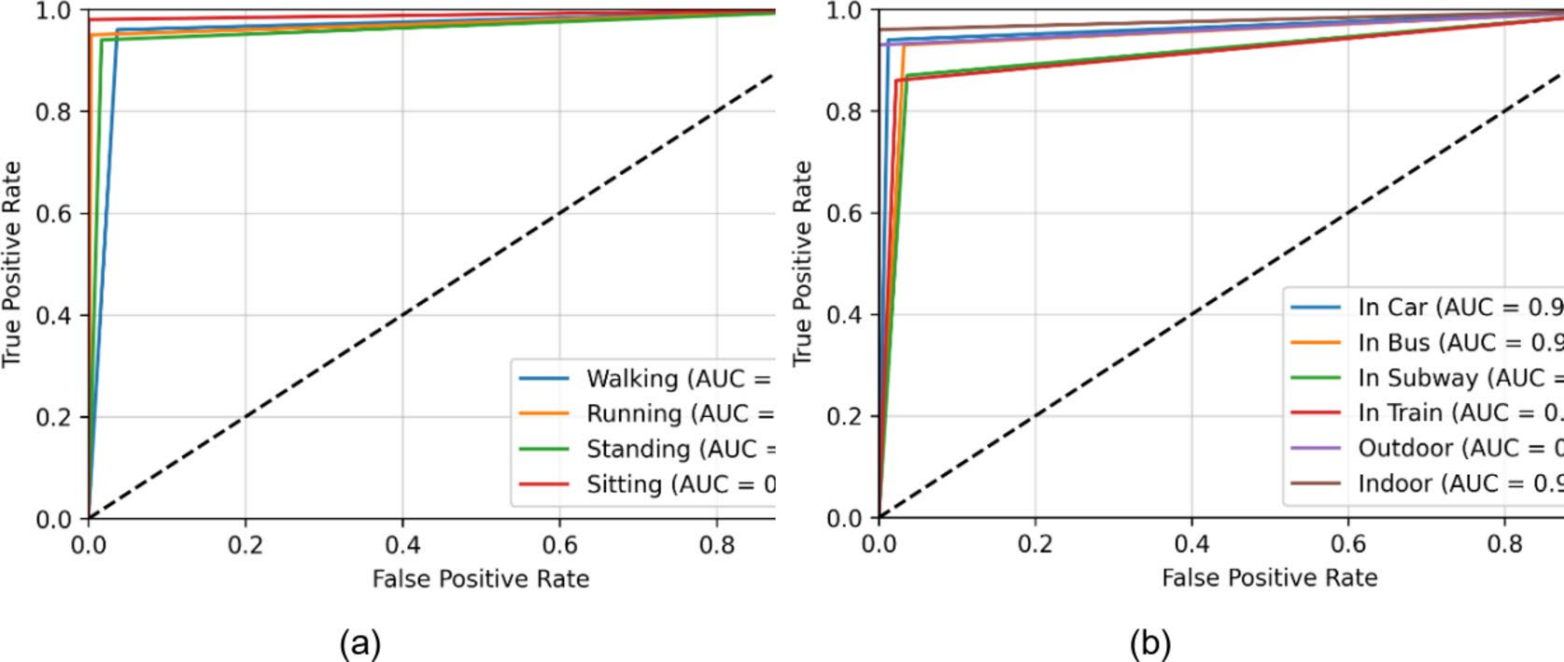
(**a**) ROC curves for HAR on SHL dataset (**b**) ROC curves for human localization on SHL dataset.

**Fig. 10:**
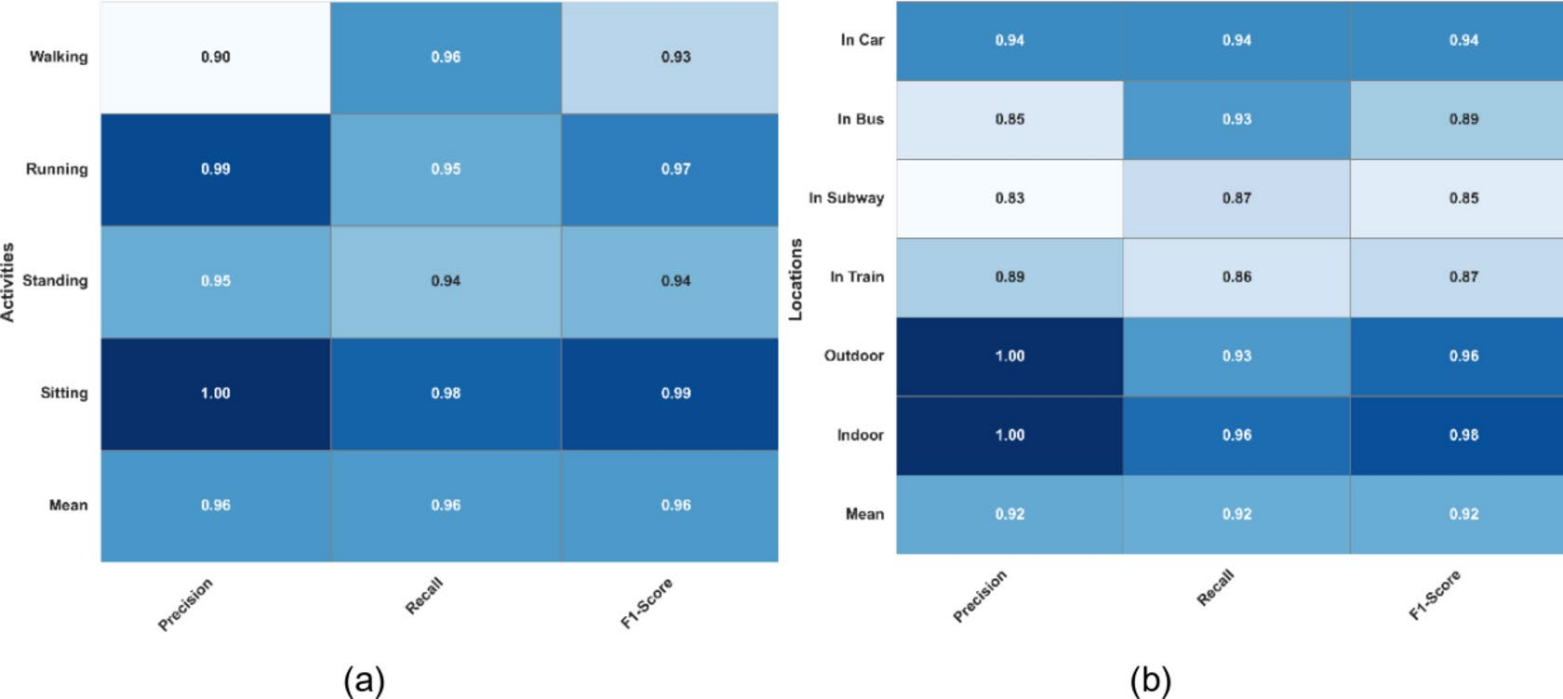
(**a**) Classification report for HAR on SHL dataset (**b**) Classification report for human localization on SHL dataset.

Similar to the Extrasensory dataset, ROC curves and classification reports were plotted for SHL dataset also. Figure 9(a) shows the ROC curves for all four activities. For all of the activities, the AUC is approaching 1 that indicates the high reliability of the proposed system for the recognition of human activities. The case is no different for the location recognition that is shown in Fig. 9(b). Additionally, the classification reports for HAR and localization are shown in 12(a) and 12(b) respectively. The mean precision and F1-score for HAR are 96% (See Fig. 10) each while for localization these performance metrices are 92% each.

### Comparison with conventional systems

Regarding the mean accuracy of activity recognition and localization, the proposed system was also compared with the previously developed state-of-the-art systems. On the Extrasensory dataset, the system’s overall activity classification accuracy was 89.25%, and its localization accuracy was 90.50%, for a mean accuracy of 89.88%. For the SHL dataset, the mean accuracy of the system was 93.62%, with activity recognition accuracy being 95.75% and location classification accuracy being 91.50%. Using the mean accuracy, the proposed system was compared with the state-of-the-art systems. The comparison in Table 3 makes it abundantly evident that the proposed solution outperformed the available state-of-the-art frameworks.

**Table 3:** Comparison of system’s performance with state-of-the-art methods.

Methods	Extrasensory	SHL
Logistic Regression^30^	0.8280	–
Random Forest^31^	0.8400	–
HAR-GCNN^32^	0.8800	–
SEED^33^	–	0.7810
CNN + PP^34^	–	0.8660
SVM^35^	–	0.9000
CNN + DNN + RNN^36^	–	0.9200
CNN^37^	–	0.9330
**RNN (Proposed)**	**0.8988** (mean [activity, location])	**0.9362** (mean [activity, location])

## Conclusion

The proposed system simultaneously addresses HAR and human localization, leveraging IoT data from smartphone and smartwatch sensors. The system denoises the data then splits up into HAR and human localization branches. Working in parallel, respective features are extracted for both of the tasks and sent to standardization pipelines where feature selection and optimization is performed. Following that, the data are transmitted to two independent RNNs for classification. The overall performance of the proposed system was better on the SHL dataset than on the Extrasensory dataset, which might be attributed to the quantity and complexity of the dataset. The system strives for high accuracy by extracting robust features and employing deep algorithm for classification. As the system utilizes IoT data from smartphone and smartwatch sensors, it expands its applicability and provides flexibility in various scenarios.

Despite deomonstrating excellent performance over both HAR and human localization, the system also have a limitation that while processing HAR and localization simultaneously, the localization results lag behind the HAR results by a few seconds which might be due to possible higher complexity of the feautres that are being extracted for localization. In the future works, we will try to overcome the lag and improve the synchronization of both outcomes.

## Data Availability

The datasets analysed during the current study are Publically available in the Extrasensory Dataset repository, http://extrasensory.ucsd.edu/. The datasets analysed during the current study are Publically available in the SHL Dataset repository, http://www.shl-dataset.org/download/.

## Code availability

The code to reproduce the results has been made available as a GitHub repository and it can be accessed at https://github.com/usm1ai/HAR-Loc-RNN.
